# Comparing survey delivery methods in healthcare: A randomized study

**DOI:** 10.1017/cts.2025.10195

**Published:** 2025-11-17

**Authors:** Gayane Tumyan, Kathleen Esselink, Ann Marie Navar, Ildiko Lingvay

**Affiliations:** 1 Division of Endocrinology, Department of Internal Medicine, UT Southwestern Medical Center, Dallas, TX, USA; 2 Office of Clinical Research, UT Southwestern Medical Center, Dallas, TX, USA; 3 Division of Cardiology, Department of Internal Medicine, UT Southwestern Medical Center, Dallas, TX, USA; 4 Peter O’Donnell Jr. School of Public Health, UT Southwestern Medical Center, Dallas, TX, USA

**Keywords:** Survey delivery methods, patient portal, research recruitment, web-based data collection

## Abstract

**Objective:**

To compare healthcare survey response rates using two widely utilized recruitment methods: email and the electronic health record (EHR) patient portal.

**Materials and methods:**

Adults with a prior history of any bariatric surgery were randomly assigned (1:1) to receive a survey invitation via email or through the EHR patient portal. A second reminder was sent using the same method. A third invitation used a crossover approach, switching to the alternate method. We compared survey completion rates, changes in research preference status, and time to survey completion. Predictors of response were assessed using multivariable logistic regression.

**Results:**

The email group had a 9.9% response rate after the first invitation and 6.5% after the second. The EHR portal group had 8.4% and 4.5% response rates, respectively. Following crossover, the third invitation yielded a 4.4% response for those switched to the EHR portal and 7.5% for those switched to email. The EHR portal group was 27% less likely to complete the survey compared to the email group. Respondents were more likely to be female, non-Hispanic, White, have a recent healthcare encounter, and have recently logged into the portal. Median time to completion was under 24 hours in both groups, with over two-thirds of responses received on the day of or the day after the invitation. A change in research preference status was observed in 2.5% of email and 4.0% of portal participants.

**Discussion and conclusion:**

Email-based recruitment yielded higher response rates than EHR portal-based recruitment, with most responses occurring shortly after invitation.

## Introduction

Surveys are an important means of large-scale data collection. Healthcare surveys are frequently utilized to assess patient experiences with care, gather feedback, and improve healthcare outcomes [1]. In medical research, surveys are essential tools as they enable researchers to gather extensive data from a specific patient population, and their use is growing [2,3].

Among the main advantages of remotely administered surveys, compared to surveys completed in the presence of a study team, are the ability to reach a wider patient population regardless of their geographical location, greater flexibility, faster recruitment/completion time, and overall low cost of testing [4,5]. However, completion rates for such remotely delivered surveys are generally very low [6].

The typical modes of delivery of healthcare surveys include secure emails, patient portals, and web applications (including social media). There are limited data comparing currently available modes of remote data collection [2,3,5–8]. Some previous studies have shown higher response rates with the patient portal compared to the email method of survey delivery, [7,8] while others have found slightly higher response rates with email delivery [9]. One study reported a 9.7% response rate among participants recruited through the patient portal, nearly double that achieved through email or postal invitations (3.5%) [7]. Another investigation found comparable response rates across patient portal (29%), mail (31.5%), and phone (32%) methods, with email lagging behind at 15.5% [8]. In contrast, another study observed a 17% response rate in the patient portal group compared with 20% for the direct email group [9].

In a randomized study, we compared two survey delivery methods and assessed their response rates, time to response, and resulting changes in research preference designation within the electronic health record (EHR) patient portal. We also evaluated the impact of subsequent reminders on response rates.

## Materials and methods

We conducted a randomized study to compare rates of survey completion when recruitment occurred through two commonly used methods of survey delivery – email or EHR patient portal.

Eligible participants were identified using an EHR (Epic Systems, Wisconsin, USA) query and were adults over 18 years old, who had not opted out of being contacted for research, and had both an email address on file and an active EHR patient portal (MyChart). The eligible population also had a history of any type of bariatric or metabolic surgery, irrespective of the time since such surgery or the facility where it was performed. Demographic info (age, ethnicity, race, sex), data on research status designation, patient portal logins, most recent healthcare encounter, and number of medications listed were obtained from EHR. All other information provided was participant-reported in the survey. A simple randomization using random number generation was performed. The study was approved by the University of Texas Southwestern Medical Center Institutional Review Board with a waiver of signed consent.

Eligible participants were randomly assigned (1:1) to receive the survey invitation either via email, which contained a link to the web-based survey (RedCap), or a notification through the EHR patient portal that allowed access to the survey within the patient portal. Survey invitations sent through the EHR patient portal message led to an automatic notification via email that a new research study message was available in the portal inbox. Participants who had the EHR mobile application installed and opted in to receive push notifications also received a mobile alert. In both cases, participants were required to log in to their EHR accounts to view and complete the survey. Invitations sent through email had a subject line that stated “Message from the UTSW Endocrinology Research Program.” Both approaches introduced the study with the same approved language.

The survey assessed people’s experience with bariatric surgery and was estimated to take less than 10 minutes to complete. Survey completion was defined as answering the first two questions.

Those who did not respond to the initial invitation within 3 weeks received a second invitation through the same recruitment method. Those who declined participation in the study, completed the survey, had an incomplete survey, or changed their research EHR portal preference designation to “do not contact” were not sent subsequent invitations. Those who had still not completed the survey within 3 weeks of receiving the second invitation received a third invitation using the alternate recruitment method: email invitations were sent to participants initially randomized to the EHR portal group, and EHR portal invitations were sent to those randomized to the email group. Participants who logged onto their patient portal account had the option to change their designated research preference (available options are undecided, opt in and opt out of research). All randomized people had an active EHR portal and an email address on file. Email bounce-backs were counted and reported in the flowchart, but subsequent attempts followed the predetermined protocol. The survey remained open (through both methods) for 60 days after the last invitation. The study was conducted from March to June of 2024.

Outcomes included overall response rates, response rates for each invitation round, time to survey completion, and changes to research preference status designations in the EHR portal during the study period.

### Statistical methods

Descriptive statistics were used to summarize demographic characteristics, response rates, and timing of responses, as well as the proportion of responses received within specific time intervals and changes in research preference designation. Continuous variables were reported as means and standard deviations (SD), and categorical variables as counts and percentages. Characteristics of responders in each group were compared using Pearson’s chi-square test for categorical variables (except race, where Fisher’s exact test was used due to small numbers within some categories) and independent samples *t*-tests for continuous variables. Predictors of survey response were assessed using multivariable logistic regression, which includes all available baseline variables and the group assignment. All statistical tests were two-sided, and a *p*-value < 0.05 was considered statistically significant without adjustment for multiple testing.

## Results

A total of 12,225 eligible people were randomized to recruitment via email (*N* = 6233) or EHR patient portal (*N* = 5992).

The eligible population had an average age of 55.7 ± 12.7 years; 83% were women, 11% were of Hispanic ethnicity, 63% were White, and 22% were African American/Black, with similar characteristics between the two groups (Table 1).

**Table 1.** Demographics of the overall eligible population

	EHR patient portal (*N* = 5992)	Email (*N* = 6233)
**Age, years**	55.6 (12.6)	55.7 (12.7)
**Ethnicity, %**		
Hispanic	10.7	11.4
Non-Hispanic	83	82.5
**Race, %**		
White	62.7	62.7
African American	22.7	22.5
Asian	0.9	0.8
Other	2.5	2.7
**Gender, %**		
Male	17	17
Female	82.9	82.9
Had an eligible* encounter in the prior 3 years, %	74.6	75.6
Time since the last encounter within the health system**, days	246.8 (296.6)	253.0 (304.0)
**Number of active medications listed in EHR, median**	12.5	12.4
**EHR patient portal login in the prior 180 days, %**	41.4%	41.7%

EHR = electronic health record.

Data are mean (standard deviation) unless otherwise noted. *Eligible encounters were Office Visits, Hospital Encounters, Video Visits, Allied Health/Nurse Visit, Ancillary Procedure, Surgery, Anesthesia Event, Procedure Visit, Infusion Visit, Research Encounter, or On-Treatment Visit; **Among those with an eligible encounter within the prior 3 years.

**Figure 1.**
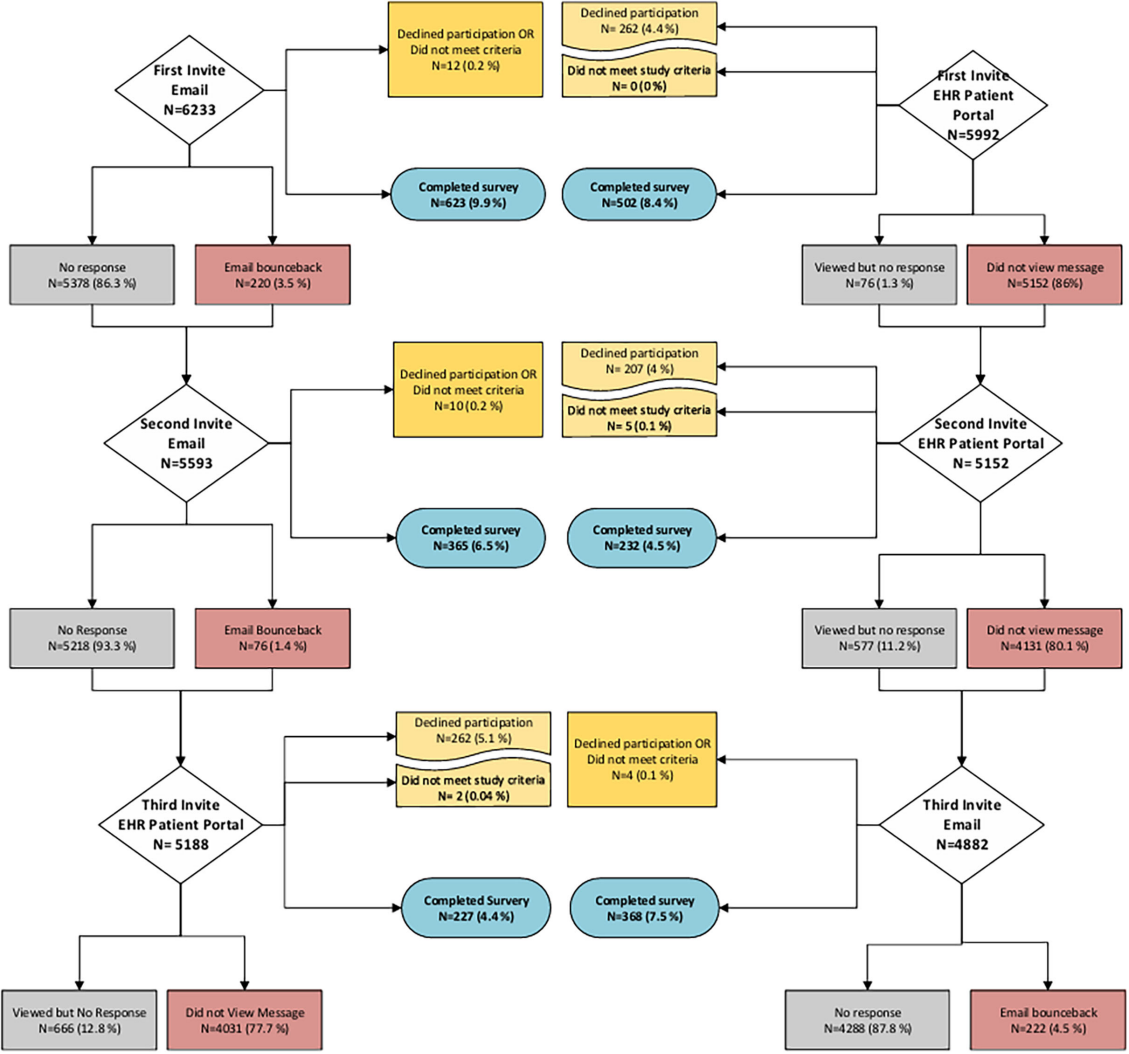
Flowchart of eligible participants through the study.

Following the first invitation, 623 people completed the survey in the email-based invitation group (9.9% response rate) and 502 in the EHR portal-based invitation group (8.4% response rate) (Figure 1). A second round of invitations was sent out to 5593 and 5152 participants via email or EHR portal, respectively, of whom 365 people completed the survey in the email-based invitation group (6.5% response rate) as compared to 232 in the EHR portal-based invitation group (4.5%). The overall response rate after the first two invites was 15.9% and 12.35% in the email and EHR patient portal groups, respectively. Of the total responses, 51.2% occurred after the first invite and 81.3% after the first two email invites; the corresponding rates were 45.5% and 66.6% in the EHR portal-based invitation group.

Table 2 presents results from multivariable modeling of factors associated with responding to the survey invitation after the first 2 invitations (delivered either via EHR patient portal or email). Participants invited through the EHR portal were less likely to respond than those invited by email (OR 0.74; 95% CI 0.66–0.82; *p* < 0.001). Female participants were more likely to respond (OR 1.25; *p* = 0.002). Compared with White participants, Black (OR 0.55; *p* < 0.001) and Asian participants (OR 0.37; *p* = 0.008) were less likely to respond, while differences for Native American participants were not significant (*p* = 0.36). Hispanic participants were also less likely to respond than non-Hispanic participants (OR 0.66; *p* < 0.001). Details on respondents in each group are presented in Table 3.

**Table 2.** Population characteristics predicting survey respondent status

Response	Odds ratio	P>|z|	[95% conf. interval]	
**EHR portal group**	0.74	0.000	0.66	0.82
**Female**	1.25	0.002	1.08	1.44
**Ethnicity**				
Hispanic	0.66	0.000	0.55	0.79
Missing ethnicity	0.84	0.183	0.64	1.09
**Race (ref: White)**				
African American or Black	0.55	0.000	0.48	0.64
Asian	0.37	0.008	0.18	0.77
Native American	0.85	0.358	0.6	1.2
Missing race	0.66	0.000	0.54	0.81
Had an eligible* encounter in the prior 3 years	1.32	0.001	1.12	1.55
**EHR patient portal login in the prior 180 days**	2.28	0.000	2.02	2.58
**Number of medications listed in EHR**	0.99	0.003	0.98	0.99

**Table 3.** Demographics of survey respondents at each recruitment attempt

	First attempt	First attempt	Second attempt	Second attempt	Third attempt	Third attempt
	**EHR patient portal** **(*N* = 502)**	**Email (*N* = 623)**	**EHR patient** **portal (*N* = 232)**	**Email** **(*N* = 365)**	**Crossed to email** **(*N* = 368)**	**Crossed to EHR patient portal** **(N = 227)**
**Age, years**	54.66 (11.95)	55.48 (12.29)	55.04 (12.82)	56.50 (12.30)	55.39 (11.98)	54.48 (11.30)
**Ethnicity, %**						
Hispanic	7.39	11.08	8.66	9.04	10.53	14.41
Non-hispanic	89.62	84.91	87.01	83.29	83.66	79.91
**Race, %**						
White	74.45	73.35	67.53	68.49	67.31	67.25
African American	18.16	15.25	23.38	16.44	18.84	18.78
Asian	0.60	0.48	0.00	0.55	1.39	0.44
Other	1.40	2.25	2.17	4.10	2.50	3.06
**Gender, %**						
Male	15.37	17.17	13.42	14.25	17.45	11.35
Female	84.63	82.83	86.58	85.75	82.55	87.77
Had an eligible* encounter in the prior 3 years, %	91.04	80.74	90.52%	78.36%	76.08	91.63%
Time since the last encounter within the health system**, days	136.97 (215.64)	211.24 (286.74)	141.98 (215.36)	204.07 (271.17)	224.83 (286.86)	133.42 (233.33)
**Number of medications listed in EHR, Mean**	13.40	12.28	13.60	12.14	12.26	13.75
**EHR patient portal login in the prior 180 days, %**	72.31	52.97	67.67	49.86	46.74	73.57

EHR = electronic health record.

Data are mean (SD) unless otherwise noted.

* Eligible encounters were Office Visits, Hospital Encounters, Video Visits, Allied Health/Nurse Visit, Ancillary Procedure, Surgery, Anesthesia Event, Procedure Visit, Infusion Visit, Research Encounter, or On-Treatment Visit. **Among those with an eligible encounter within the prior 3 years.

The third invitation was sent out using the alternate approach method (email-based invitation for those originally in the EHR patient portal group and vice versa). Overall, 227 (4.4%) people who received the third invite through the EHR portal completed the survey as compared to 368 (7.5%) of those who received it through email (Figure 1). The response rate after all three attempts was 19.49% among those randomized to the email group (but received a third invite by EHR portal) and 18.39% among those randomized to the EHR portal group (with a third invite by email).

**Figure 2.**
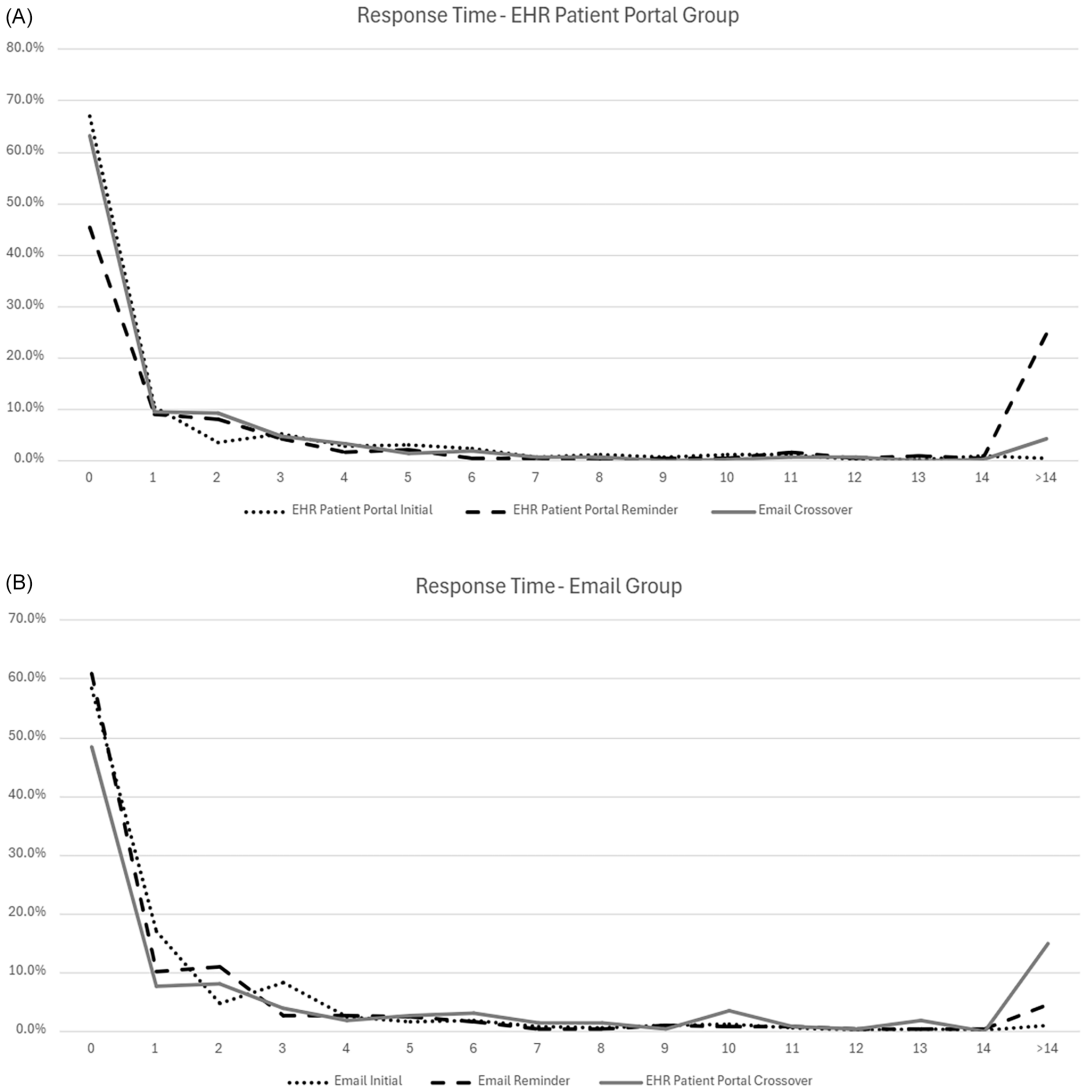
Time to survey completion after each recruitment attempt in the EHR patient portal group (A) and email group (B). Those who changed their research participation preferences, died, or started the survey but did not answer any questions were not included in subsequent invitations. EHR = electronic health record.

Figure 2 shows response times for participants invited to participate after each invitation. Most surveys were completed within the first 24 hours of receiving the invitation. In the email group, 58.4% of responses occurred within the first 24 hours of the first approach, while in the EHR portal-based group, 67.1% of responses occurred within 24 hours. After the first round of invitations, 94.9% of responses were received within the first week in the email-based group, compared to 94.7% in the EHR portal-based group. Following the second attempt, 60.9% and 45.3% of responses occurred in the first 24 hours in the email group and EHR patient portal group, respectively. Following the second round of invitations, 91.8% and 71.7% of responses occurred within the first week in the email-based group and EHR patient portal group, respectively. Upon the third invite (cross-over), 63.3% of responses occurred within 24 hours when the EHR-based group was switched to email invite, and 48.5% when the email group was switched to EHR portal-based survey delivery. After the third invitation was sent using the alternate contact method, 76.9% of responses were received within the first week in the email-based group and 93.7% in the EHR portal-based group.

Most patients who received a survey invitation did not update their portal preference regarding permission to be contacted for research studies: 1.6% of those in the email group and 2.7% of those in the EHR portal group changed their status to “Do Not Contact” after being sent the invitation (Table 4).

**Table 4.** Changes to research preference status designation in the EHR patient portal by randomized group

	“Ok to contact” to “Undecided”	“Ok to contact” to “Do not Contact”	“Undecided” to “Ok to contact”	“Undecided” to “Do not contact”	No change
**Email group (*N* = 6219)**	34 (0.5)	12 (0.2)	20 (0.3)	90 (1.4)	6063 (97.5)
**EHR patient portal group (*N* = 5989)**	54 (0.9)	21(0.3)	26 (0.4)	141 (2.4)	5747 (96.0)

EHR = electronic health record.

Data are *N* (%).

## Discussion

In this randomized study of two recruitment methods for a healthcare survey, we found that recruitment by email resulted in higher response rates compared to recruitment via the EHR patient portal (15.9% vs 12.3%, respectively). The response rate in the EHR patient portal group subsequently improved when a third invite was delivered via email, while the reverse occurred when a third invite was delivered via the EHR portal for those initially randomized to email invite. Most surveys were completed within 24 hrs of being received. Survey responders were more likely to be of White ethnicity, have female gender, and have interactions with the healthcare system within the prior 3 years and EHR portal use within 180 days.

There are a few possible explanations for the observed higher response rate for email-based recruitment compared to the EHR patient portal recruitment. First, accessing the survey may have been more difficult for the study participants in the EHR patient portal group as it required login information and a two-step process to reach the survey within the portal. Additionally, some patients might not have the technical skills to use patient portal applications but have greater familiarity with the use of email. Furthermore, in today’s healthcare system, patients often receive numerous notifications via the EHR patient portal, such as immunization reminders and appointment check-ins, which could contribute to “alert fatigue.” This phenomenon has been previously described among physicians receiving clinical trial alerts [10].

Prior studies demonstrated that ease of access is a critical determinant of survey participation. Electronic survey methods tend to perform better among younger, digitally engaged participants but yield lower response rates among older adults, indicating that familiarity with technology and ease of navigation affect engagement [8]. Similarly, higher recruitment has been observed through patient portal messaging compared with email or mail when participants are already active portal users, highlighting that a familiar and easily accessible platform can enhance participation [7]. Other investigations have also found that respondents to web-based surveys are typically younger and more often female [11,12]. In our study, female participants were more likely to complete the survey (OR 1.25, *p* < 0.02), while age was not significantly associated with response. As compared to the rest of our study population, respondents tended to have a shorter time since the last encounter with the healthcare system, and over 60% used the EHR portal within 180 days before randomization. Not unexpectedly, responders to the EHR portal invite were significantly more likely than responders to the email invite to have recent healthcare encounters, having had logged in to the EHR portal within the prior 180 days, and have a higher number of active medications listed – all reflecting that survey invites through this method are more likely to be successful among those with a higher use of the healthcare system and therefore more familiar with the EHR patient portal. Therefore, an EHR-based invite might be desirable for surveys that target a population with a higher disease burden, while an email-based invite might be more suitable for a more general population with a lower use of the healthcare system.

With more than 12,000 participants, our study cohort is substantially larger than those in earlier investigations (which enrolled roughly 800 – 6600 participants). Its randomized and crossover design (email ↔ portal) reduces selection and access bias, enabling a clearer assessment of how the delivery method itself – rather than participant characteristics – drives response differences.

Our study also provides important insights regarding best practices for using both the EHR patient portal and emails to invite participants for surveys. First, we found that sending a second invitation to non-responders substantially increased participation rates for both those sent EHR and email-based invitations. This further increased when a third message was sent via a different channel, suggesting that at least three messages should be sent to each potential participant to maximize response rates. We did not continue to send messages to avoid overburdening potential respondents, so we cannot determine at what point sequential invitations lead to diminishing returns for enrollment. Second, we found that the vast majority of those who will respond do so within 1 day, and nearly all who will respond to a message do so within a week, suggesting that longer wait times between messages may not be necessary. The fact that completion rates are highest in the first 24 hours after sending the messages raises the important question regarding what day of the week or time of day may be most impactful to maximize engagement, a research question that should be explored in future studies.

In this study, 3% of participants modified their research designation status after receiving the survey invite. The change in research self-designation status is likely influenced by multiple factors. It is possible that being contacted about a research study prompted patients to re-evaluate their preferences regarding being contacted for future studies. Additionally, some patients may have noticed their research designation status for the first time after being contacted for a study. Those randomized to the EHR patient portal group were more likely to change their research designation status in the EHR patient portal, a finding likely explained by the fact that their research preferences are displayed in the same area of the portal as the research invite. Additionally, alert fatigue resulting from frequent EHR portal notifications may have influenced their decision to change their research designation as well. Updating research designation status is a positive outcome, as it provides a current reflection of their willingness to participate in research and ensures they are not approached if they prefer not to be.

This experiment allowed us to reflect on the advantages and drawbacks of each approach used. The EHR patient portal offers a more secure, fully trackable experience at a higher cost and requires specialized resources for setup and data extraction. On the other hand, data collection via email leads to a higher response rate and may be easier to set up for institutions that do not regularly use bulk EHR portal messaging, thus could be deployed faster. However, email invites cannot be tracked to see if they were viewed, and given that they contain a web-based link, they can be sorted into the junk folder or blocked by certain servers.

### Limitations

Our study has limitations. First, it was conducted in a single academic center with a high overall EHR utilization rate (>70%) on a specialized population (post-bariatric/metabolic surgery); therefore, findings should be reproduced in other healthcare settings and different populations. There was a higher proportion of female patients (83%), dictated by the research question of the survey and the population it applied to. This limits the generalizability of our findings to other clinical populations or broader demographic groups. Another consideration is that the content of the survey itself, focused on bariatric surgery, may have impacted participants’ likelihood of responding, independent of the delivery method. We did not have access to participants’ bariatric surgery dates, which could have provided insight into whether the time since surgery influenced engagement with the survey. Future studies should evaluate whether the timing of survey distribution relative to a major health event affects response rates.

Participants were randomly assigned to one of the two methods of survey invitation. No data was collected on preference for communication or comfort with technology, making it hard to discern whether response differences were due to delivery method or user comfort. Future research could explore whether offering both options to the entire study population affects which method they choose, what factors might influence their decision, and how this might impact participant engagement. Additionally, no formal cost analysis was conducted between the two delivery methods.

## Conclusion

Medical survey delivery through email generated higher response rates than delivery via the EHR patient portal. Most survey responses were received within the first 24 hours of each recruitment attempt. Participants in the EHR portal group were more likely to update their research designation compared to those in the email group. These findings should inform survey delivery methods and approaches that enhance health survey completion rates.
